# Degenerative tendon matrix induces tenogenic differentiation of mesenchymal stem cells

**DOI:** 10.1186/s40634-023-00581-4

**Published:** 2023-02-14

**Authors:** Joongwon Hwang, Seung Yeon Lee, Chris Hyunchul Jo

**Affiliations:** 1 Department of Translational Medicine, College of Medicine, Seoul National University, Daehak-Ro 103, Jongno-Gu, Seoul, 03080 South Korea; 2 Department of Orthopedic Surgery, College of Medicine, SMG-SNU Boramae Medical Center, Seoul National University, 20 Boramae-Ro 5-Gil, Dongjak-Gu, Seoul, 07061 South Korea

**Keywords:** Degenerative tendon matrix, Extracellular matrix, Tenogenic differentiation, Mesenchymal stem cells, Senescence

## Abstract

**Purpose:**

Mesenchymal stem cells (MSCs) react dynamically with the surrounding microenvironment to promote tissue-specific differentiation and hence increase targeted regenerative capacity. Extracellular matrix (ECM) would be the first microenvironment to interact with MSCs injected into the tissue lesion. However, degenerative tissues would have different characteristics of ECM in comparison with healthy tissues. Therefore, the influence of degenerative ECM on tissue-specific differentiation of MSCs and the formation of matrix composition need to be considered for the sophisticated therapeutic application of stem cells for tissue regeneration.

**Methods:**

Human degenerative tendon tissues were obtained from patients undergoing rotator cuff repair and finely minced into 2 ~ 3 mm fragments. Different amounts of tendon matrix (0.005 g, 0.01 g, 0.025 g, 0.05 g, 0.1 g, 0.25 g, 0.5 g, 1 g, and 2 g) were co-cultured with bone marrow MSCs (BM MSCs) for 7 days. Six tendon-related markers, scleraxis, tenomodulin, collagen type I and III, decorin, and tenascin-C, osteogenic marker, alkaline phosphatase (ALP), and chondrogenic marker, aggrecan (ACAN), were analyzed by qRT-PCR. Cell viability and senescence-associated beta-galactosidase assays were performed. The connective tissue growth factor was used as a positive control.

**Results:**

The expressions of six tendon-related markers were significantly upregulated until the amount of tendon matrix exceeded 0.5 g, the point where the mRNA expressions of all six genes analyzed started to decrease. The tendon matrix exerted an inhibitory effect on ACAN expression but had a negligible effect on ALP expression. Cell viability did not change significantly over the culture period. The amount of tendon matrix exceeding 0.01 g significantly increased the SA-βgal activity of BM MSCs.

**Conclusion:**

This study successfully demonstrated tendon ECM-stimulated tenogenesis of BM MSCs through an indirect co-culture system without the use of exogenous growth factors and the alteration of cellular viability. In contrast to the initial hypothesis, the tenogenesis of BM MSCs induced with the degenerative tendon matrix accompanied cellular senescence.

## Background

Tendinopathy, characterized by chronic degeneration and impaired function of a tendon, is one of the most prevalent musculoskeletal diseases [19, 29]. Due to the predominantly degenerative characteristic of the disease, conventional therapies for tendinopathy, including classical physiotherapy, anti-inflammatory drugs, and corticosteroid injections, are neither effective nor evidence-based [7, 19, 29]. However, advances in the field of regenerative medicine have shown positive results of mesenchymal stem cells (MSCs) on tendon regeneration based on their functions as connective tissue cell progenitors and potent immunomodulators [7, 38]. Accumulating evidence suggests that MSCs react dynamically with the surrounding microenvironment to promote tissue-specific differentiation and hence trigger their immunomodulatory responses required for targeted tendon healing [20, 32].

Extracellular matrix (ECM), a complex orchestration of macromolecules composed of over 300 proteins, would be the first microenvironment to interact with MSCs injected into the tissue lesion [5, 28]. Although the fundamental mechanisms of how the therapeutic application of stem cells contributes to tissue regeneration are largely unknown, numerous studies have reported that ECM isolated from a particular tissue promoted tissue-specific differentiation of stem cells [1, 9, 24, 25, 27, 35]. However, patients with tendinopathy do not retain the normal characteristics of ECM. Rather, ECM interacting with stem cells is under progressive degeneration due to a change in cell function and consequent alteration of both matrix composition and organization resulting in a mechanically weaker tendon [2, 4, 15]. Previous studies regarding the effects of degenerative osteoarthritic ECM on tissue-specific chondrogenesis of MSCs have produced conflicting results [9, 12, 22]. Therefore, the influence of degenerative tendon matrix on tissue-specific differentiation of MSCs and the formation of matrix composition need to be accounted for sophisticated evaluation of the therapeutic application of MSCs for tissue regeneration.

The purpose of this study was to investigate the effects of the degenerative tendon matrix on the differentiation, viability, and senescence of bone marrow MSCs (BM MSCs). We hypothesized that the degenerative tendon matrix could induce tenogenic differentiation of MSCs without the use of exogenous growth factors and the alteration of cellular viability and senescence. To test the hypothesis, tendon tissues were harvested from patients undergoing rotator cuff repair, finely minced into 2 ~ 3 mm fragments, and co-cultured with BM MSCs using 3-μm pore size transwells for up to 7 days. Tenogenic, osteogenic, and chondrogenic markers were analyzed, and the influence of the ECM on the cellular senescence of BM MSCs was assessed.

## Materials and methods

### Tissue samples

The study protocol was approved by the Institutional Review Board at our institution and was conducted in accordance with the approved guidelines (Seoul National University Boramae Medical Center IRB No. 20120405/06–2012-78/118). All patients from whom tissue specimens were harvested provided informed consent. Human tendon tissues (*n* = 15) and bone marrow were obtained from patients undergoing arthroscopic rotator cuff repair. As previously described [17], the tendon grade was assessed on the basis of gross appearance at the time of surgery with respect to three criteria: (1) fraying over half the tendon thickness, (2) delamination, and (3) thinning of less than half the thickness of the normal rotator cuff. Gross tendon quality was graded as A if none of these criteria were met, B if fraying or delamination was identified, and C if both fraying and delamination or thinning with or without the other two criteria were identified. Harvested human tendon tissues were frozen and stored at -80 °C until further use. Demographic characteristics of harvested tendon tissues are described in Table 1.

**Table 1:** Participant demographics from the current study

Characteristic	Tendon (*n* = 15)
Mean age, y	65.47 ± 7.15
Sex (male: female), n	6: 9
Anteroposterior size, mm	29.00 ± 10.40
Mediolateral size, mm	20.00 ± 7.34
Tendon grade (A: B: C), n	6: 7: 2
Global Fatty Degeneration Index	1.93 ± 0.34

### Isolation and culture of BM MSCs

BM MSCs were isolated as previously described [18]. Extracted bone marrow samples (*n* = 9) from 5 males and 4 females were diluted twice with calcium- and magnesium-free phosphate-buffered saline (DPBS) and layered on top of Ficoll-PaqueTM Premium (GE Healthcare, Uppsala, Sweden) at a ratio of 1:2. This was then centrifuged at 400 g (with brake off) for 30 min at 20 °C. The uppermost layer was aspirated and discarded. The mononuclear layer was collected and diluted three times with DPBS. This was centrifuged at 400 g for 5 min and washed again with DPBS. The supernatant was discarded, and the pellet was resuspended with 10 mL of growth medium (low glucose Dulbecco’s Modified Eagle’s Medium (DMEM) containing 10% heat-inactivated fetal bovine serum (FBS), 100 U/mL penicillin, and 100 μg/ml streptomycin). This was then centrifuged at 400 g for 5 min. The cellular pellet was resuspended in a growth medium. The cells were seeded onto conventional tissue culture plates at a concentration of 1 × 10^6^ cells/cm^2^ and incubated in a 5% CO^2^ incubator with humidified air at 37 °C. The medium was replaced every 3 days. When cells reached 80% confluence, they were split at a ratio of 1:4. BM MSCs at passage 3 (P3) were used for all experiments.

### Colony-forming-unit fibroblasts (CFU-F) assay

CFU-F assays were performed by using a previously described method [16]. Briefly, the cells isolated from BM (*n* = 1) were seeded on the bottom of 6-well culture plates at a density of 2 × 10^4^ and 4 × 10^4^ cells/cm^2^ and cultured for 14 days. After 14 days, cells were washed twice with DPBS and fixed in 4% paraformaldehyde for 20 min, stained with 0.1% crystal violet (Sigma Aldrich) for 1 h, and then rinsed with tap water. Aggregates of 50 cells or more with a fibroblast phenotype were defined as a CFU-F colony. The experiment was performed in triplicate.

### Flow-cytometry

To confirm that the MSCs met the defining immunophenotype outlined by the International Society for Cellular Therapy (ISCT), the surface phenotypes of human BM MSCs (*n* = 3) were analyzed by flow cytometry as previously described [16]. Briefly, a total of 5 antibodies were used in flow cytometry: CD45, CD73, CD90, CD105, and HLA-DR (Becton Dickinson). After cells had been detached, aliquots of 5 × 10^5^ cells at P3 were washed twice with DPBS, centrifuged, washed in ice-cold DPBS supplemented with 1% bovine serum albumin (FCM buffer), and fixed in 2% paraformaldehyde in FCM buffer, followed by incubation with fluorescein isothiocyanate (FITC)- or phycoerythrin (PE)-conjugated antibodies for 15 min on ice in a dark room. Data were obtained by analyzing 10,000 events on a Becton Dickinson FACSAria with FACSDiva software (Becton Dickinson, San Jose, Calif., USA). All analyses were standardized against negative control cells incubated with isotype-specific IgGs.

### Indirect co-culture of tendon matrix and BM MSCs

The human BM MSCs (*n* = 3) were seeded on the bottom of 6-well culture plates at a density of 4 × 10^3^ cells/cm^2^ and were allowed to attach for 24 h in DMEM supplemented with 10% heat-inactivated FBS and antibiotic solution (100 U/mL penicillin and 100 μg/ml streptomycin). Meanwhile, human tendon tissues were slowly thawed at 4 °C. Tendon tissues were washed twice in DPBS and finely minced into 2 ~ 3 mm pieces. Collected tendon fragments were placed on each 3-μm pore size transwells. BM MSCs and tendon fragments were co-cultured for up to 7 days. The medium was changed every 2 to 3 days. The BM MSCs without tendon fragments were used as a negative control and connective tissue growth factor (CTGF) was used as a positive control. All experiments were performed in duplicate.

### RNA extraction and quantitative RT-PCR analysis

Total RNA was extracted, and reverse transcription and amplification were performed as previously described [16]. Briefly, total RNA was extracted using the RNeasy® mini kit (QIAGEN, Hilden, Germany) according to the manufacturer’s protocol and quantified using a Nano-Drop ND-100 spectrophotometer (NanoDrop, Wilmington, DE, USA). Then, 1 μg of mRNAs was converted to complementary DNA (cDNA) by Superscript III Reverse Transcription kit (Invitrogen, Carlsbad, CA, USA). Synthesized cDNA was diluted to 300 μL with PCR-grade water and then stored at -20 °C until further use.

RT-PCR was performed by utilizing a LightCycler® 480 (Roche Applied Science, Mannheim, Germany). TaqMan® Gene Expression Assays (Applied Biosystems, Foster City, CA, USA) was used as a probe/primer set. The PCRs were performed in a final volume of 20 μL containing 10 μL 2 × LightCycler® 480 Probes Master (Roche Applied Science), 1 μL TaqMan® Gene Expression Assay (Applied Biosystems), 5 μL cDNA as the template, and 4 μL PCR grade water using the following program: 95 °C for 10 min, 40 cycles of 95 °C for 10 s and 60 °C for 1 min, followed by 72 °C for 4 s, and a final cooling at 40 °C for 30 s. Experiments were performed in duplicate, and averaged values were calculated for normalized expression levels. During PCR amplification, amplified product amounts were monitored by continuous measurement of fluorescence. mRNA levels were normalized versus GAPDH as follows; the cycle number at which the transcript of each gene was detectable (threshold cycle, Ct) was normalized against Ct of GAPDH, which is referred to as ΔCt. mRNA levels relative to GAPDH are expressed as 2^−ΔCt^, where ΔCt = C_T gene of interest_ – C_T GAPDH_. A total of eight gene-specific primers sorted into four categories are used: (1) tenogenic markers: scleraxis (SCX), tenomodulin (TNMD); (2) matrix molecules: collagen type I (Col I) and III (Col III), decorin (DCN), tenascin-C (TNC); (3) osteogenic marker: alkaline phosphatase (ALP); (4) chondrogenic marker: aggrecan (ACAN).

### Cell viability assay

To analyze the cell viability of BM MSCs, WST-1 assay was performed after indirect co-culture with tendon matrix. The human BM MSCs (*n* = 3) were seeded on the bottom of 24-well culture plates at a density of 4 × 10^3^ cells/cm^2^ and were allowed to attach for 24 h in low glucose DMEM supplemented with 10% heat-inactivated FBS and antibiotic solution (100 U/mL penicillin and 100 μg/ml streptomycin). The tendon matrix with different weights was then placed on the cell culture inserts of each well. Cell viability was determined using the EZ-Cytox cell viability assay kit (Daeil Lab Service, Seoul, Republic of Korea) according to the manufacturer’s instructions on day 7 of the co-culture. Each well was washed with DPBS, 200 µL of the 1:10 WST-1 agent diluted to the culture media was added and cultured for an hour, and 100 µL of each well was transferred to a 96-well plate to read at a 450 nm wavelength using a spectrophotometer.

### Senescence-associated beta-galactosidase (SA-βgal) assay

To analyze the cellular senescence of BM MSCs, SA-βgal assay was performed after indirect co-culture with the tendon matrix. The human BM MSCs (*n* = 6) were seeded on the bottom of 24-well culture plates at a density of 4 × 10^3^ cells/cm^2^ and were allowed to attach for 24 h in low glucose DMEM supplemented with 10% heat-inactivated FBS and antibiotic solution (100 U/mL penicillin and 100 μg/ml streptomycin). The tendon matrix with different weights was then placed on the cell culture inserts of each well. Cellular senescence was determined using the β-galactosidase staining kit (Cell Signaling Technologies, Danvers, MA, USA) according to the manufacturer’s protocol on day 7 of the co-culture. Senescent BM MSCs were determined by the blue color precipitate over the cells using light microscopy, and images were captured.

### Statistical analysis

All data values were expressed as means and standard deviations. The significance of the statistical difference was determined using 1-way analysis of variance with post hoc analysis of the Bonferroni multiple comparison test. The analysis was performed using SPSS software, version 13.0 (SPSS Inc, Chicago, IL, USA), and *p* < 0.05 was considered to be statistically significant.

## Results

### Phenotypic characterization of BM MSCs

**Fig. 1:**
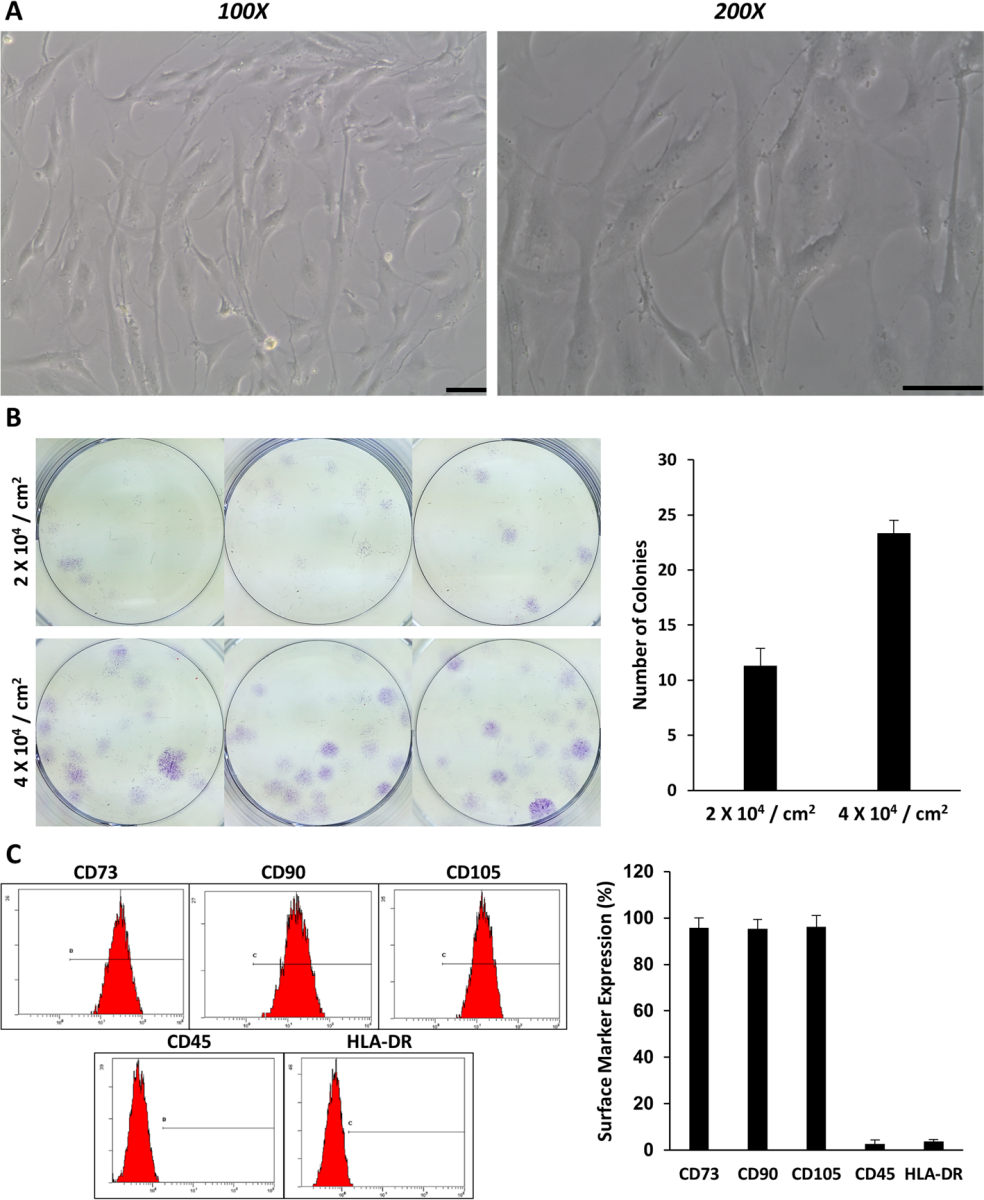
Phenotypic characterization of BM MSCs. **A** Morphology of human BM MSCs (original magnification: 100 × and 200x, scale bar = 100 μm). **B** CFU-F assay of human BM MSCs with two different initial seeding cell densities and its semi-quantification. **C** Immunophenotype expression analysis of human BM MSCs using flow cytometry

The isolated human BM MSCs were observed under an optical microscope and the image in Fig. 1A-B showed that the cells exhibited representative MSC morphology with spindle- and fibroblast-like shapes accompanied by colony-forming features. Moreover, the data from the flow cytometer assay (Fig. 1C) revealed that the isolated cells highly expressed the positive mesenchymal markers (CD73, CD90, and CD105) and presented low levels of hematopoietic markers (CD45 and HLA-DR). Taken together, analyzed BM MSCs met the defining immunophenotype outlined by the ISCT [11].

### Effects of human degenerative tendon matrix on gene expressions of tenogenic markers and matrix molecules of human BM MSCs

**Fig. 2:**
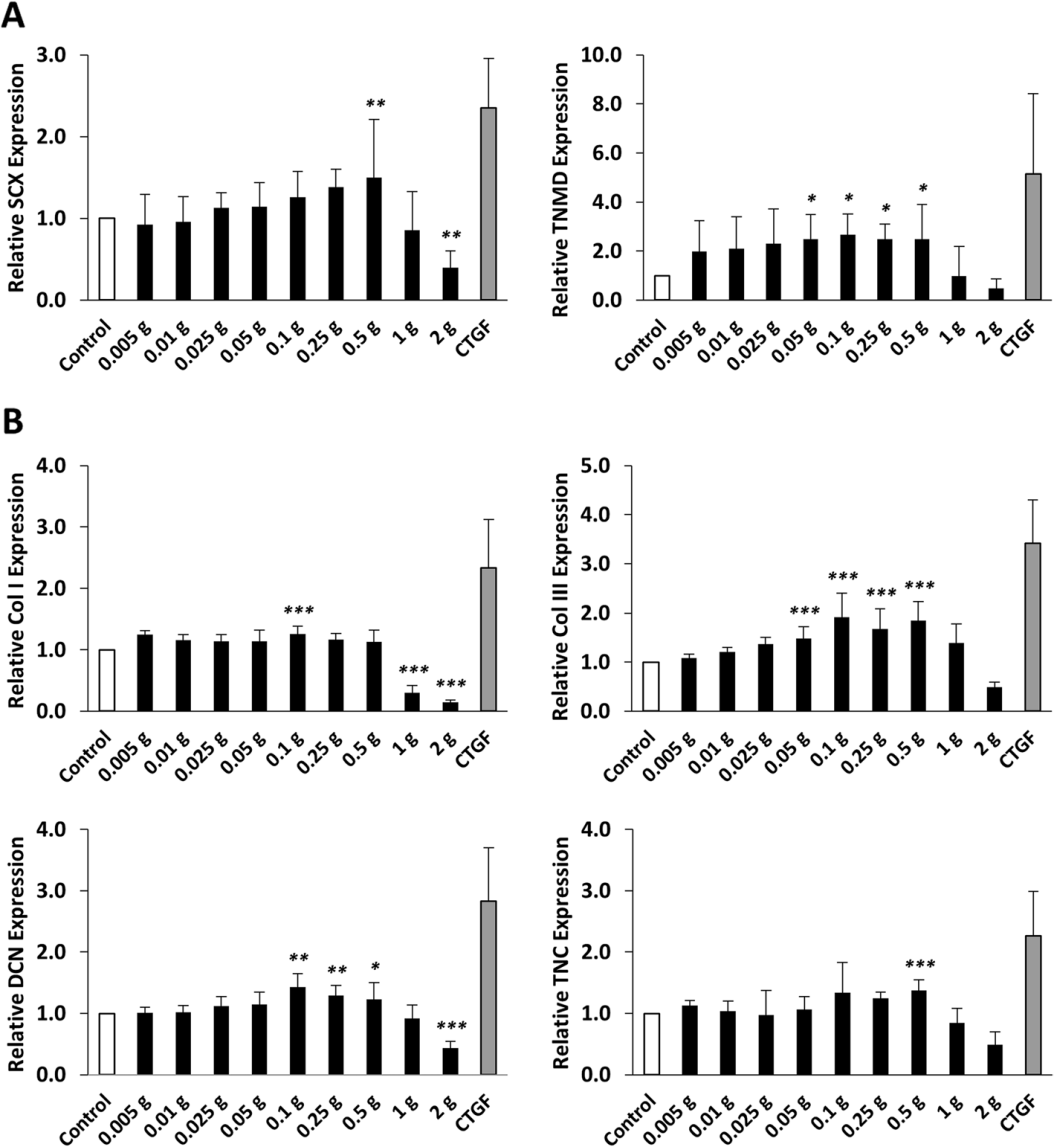
Effects of human degenerative tendon matrix on gene expression of six tendon-related markers. Relative mRNA expression of (**A**) two tenogenic markers, including SCX and TNMD, and (**B**) four matrix molecules, including Col I, Col III, DCN, and TNC, was detected by qRT-PCR. *p < 0.05, **p < 0.01, and ***p < 0.001

To evaluate the effect of human degenerative tendon matrix on tenogenic differentiation and matrix formation of BM MSCs, relative mRNA expression of two tenogenic markers (SCX and TNMD) and four matrix molecules (Col I, Col III, DCN, and TNC) were measured by quantitative RT-PCR after 7 days of co-culture. The results in Fig. 2 showed a dose-dependent tendency between the amount of the tendon matrix and the relative mRNA expression of the analyzed genes. The expressions of six tendon-related markers were significantly upregulated until the amount of tendon matrix exceeded 0.5 g, the point where the mRNA expressions of all six genes analyzed started to decrease. CTGF significantly increased the expression of all six genes analyzed. Collectively, the above results revealed that adequate amounts of the degenerative tendon matrix could promote while excessive amounts could inhibit the tissue-specific tenogenesis of BM MSCs.

### Effects of human degenerative tendon matrix on gene expressions of osteogenic and chondrogenic markers of human BM MSCs

**Fig. 3:**
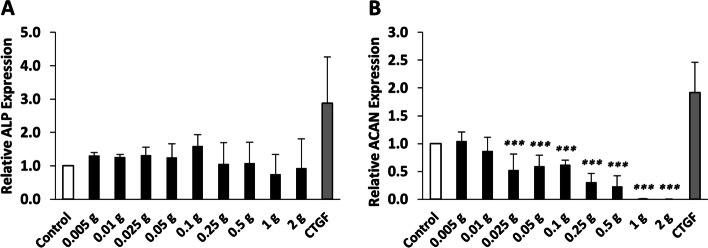
Effects of human degenerative tendon matrix on gene expressions of osteogenic and chondrogenic markers of human BM MSCs. Relative mRNA expression of (**A**) osteogenic marker, ALP, and (**B**) chondrogenic marker, ACAN, was detected by qRT-PCR. ***p < 0.001

To gain further insight into the effects of the degenerative tendon matrix on tissue-specific differentiation of BM MSCs, relative mRNA expressions of the osteogenic marker, ALP, and the chondrogenic marker, ACAN, were measured. As shown in Fig. 3A, the degenerative tendon matrix had no significant effect on the expression of ALP. However, as shown in Fig. 3B, 0.025 g, 0.05 g, 0.1 g, 0.25 g, 0.5 g, 1 g, and 2 g of tendon matrix significantly downregulated the expression of ACAN by 0.52-, 0.59-, 0.62-, 0.31-, 0.23-, 0.01-, and 0.001-fold, respectively. CTGF significantly increased the expression of ALP and ACAN. In brief, these results revealed that the degenerative tendon matrix could have an inhibitory effect on chondrogenic differentiation with negligible effect on osteogenic differentiation of BM MSCs.

### Effects of human degenerative tendon matrix on viability and cellular senescence of human BM MSCs

**Fig. 4:**
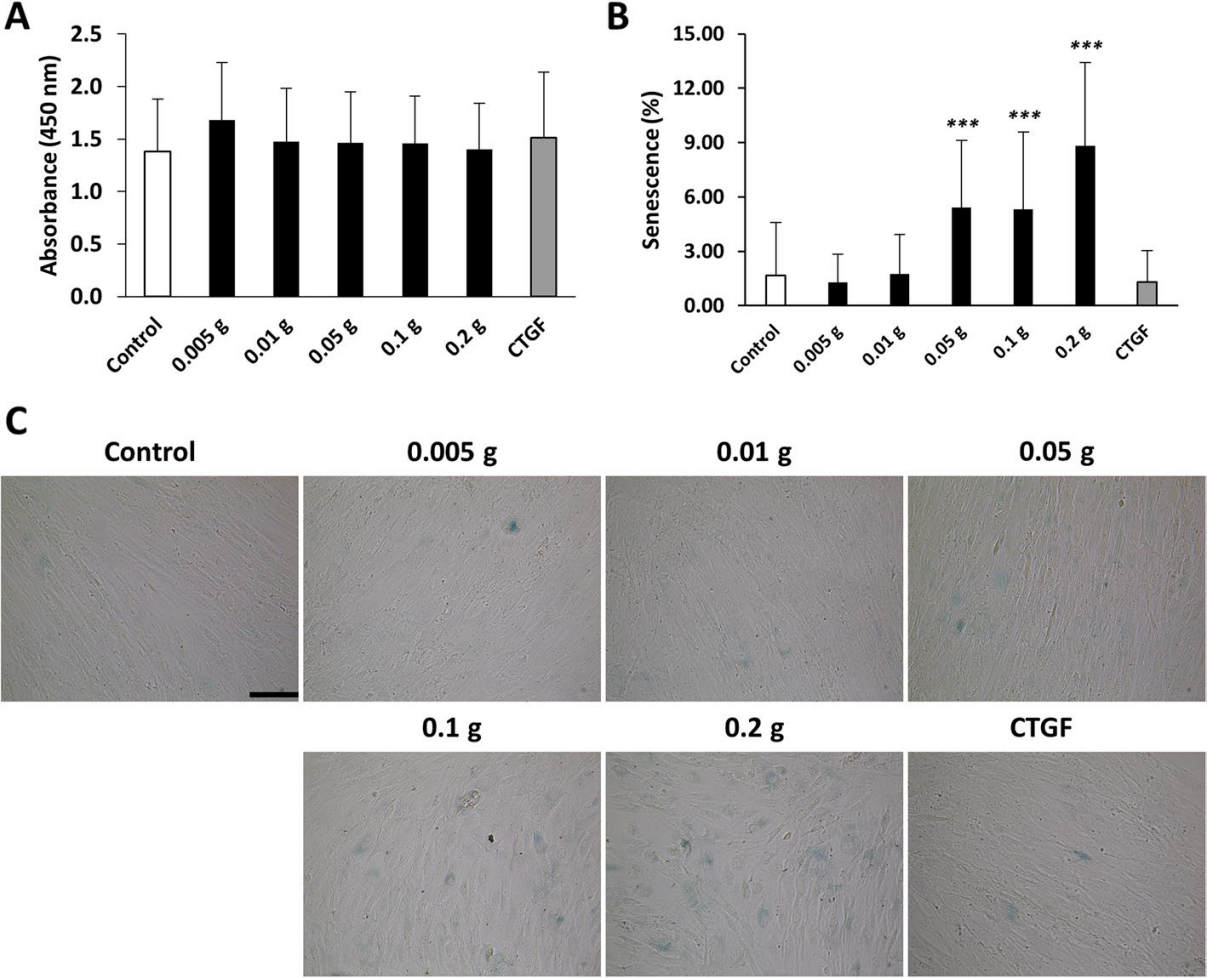
Effects of human degenerative tendon matrix on viability and senescence of human BM MSCs. **A** Measurement of cell viability using EZ-Cytox cell viability assay kit. **B** Measurement of cellular senescence using senescence β-galactosidase staining kit. **C** Morphology of human BM MSCs after β-Galactosidase Staining (original magnification: 100x, scale bar = 200 μm). ***p < 0.001

To further investigate the effects of degenerative tendon matrix on cytotoxicity and senescence of BM MSCs, cell viability and SA-βgal assays were performed. As shown in Fig. 4, the results demonstrated that the human degenerative tendon matrix did not significantly change the viability of BM MSCs regardless of different amounts of tendon matrix. In the case of cellular senescence, degenerative tendon matrix of 0.05 g, 0.1 g, and 0.2 g significantly upregulated SA-βgal activity of BM MSCs by 3.18-fold (5.41%), 3.12-fold (5.31%), and 5.18-fold (8.83%), respectively. CTGF exerted insignificant effects on both the cell viability and the SA-βgal activity. Taken together, these data suggested that a certain amount of the degenerative tendon matrix induced cellular senescence without affecting cytotoxicity.

## Discussion

The most important findings of this study are (1) human degenerative tendon matrix of 0.005 g, 0.01 g, 0.025 g, 0.05 g, 0.1 g, and 0.5 g dose-dependently induced tenogenesis of BM MSCs with upregulations of tenogenic markers, SCX and TNMD, and matrix molecules, Col I, Col III, DCN, and TNC, until the tendon matrix of 1 g and 2 g inhibited the tenogenesis with downregulations of abovementioned tendon-related markers; (2) the tenogenesis of BM MSCs was tissue-specific as the degenerative tendon matrix exerted an inhibitory effect on chondrogenesis of BM MSCs with downregulated ACAN expression and had a negligible effect on osteogenesis of BM MSCs with unchanged ALP expression; (3) an increase in the amount of tendon matrix accompanied a rise in SA-βgal activity without affecting the cytotoxicity. These results suggest that an adequate amount of degenerative tendon matrix alone could induce tenogenesis of BM MSCs through an indirect co-culture system without altering cellular viability.

The results of our study revealed that the human degenerative tendon matrix upregulated relative mRNA expression of tenocyte-specific markers of SCX, TNMD, Col I, Col III, DCN, and TNC. As a specific marker for not only tendon/ligament progenitor but also differentiated cells induced at the earliest stage of tendon/ligament lineage specification, SCX is highly expressed during tenogenesis and persists through differentiation, affecting both cellular differentiation and ECM organization [23]. TNMD is a type II transmembrane protein specifically expressed in hypovascular connective tissues such as the tendon and ligament that serves as a phenotypic marker for tendon development [26, 34]. BM MSCs have not been reported for expression of TNMD unless they are appropriately stimulated [24]. Col I, the most abundant form of collagen in the tendon and throughout the body, provides the tendon with its mechanical durability and strength [6]. Col III, involved in the healing process in the tendon, is another fibrillar collagen that is closely linked to type I collagen [6]. DCN, the most abundant proteoglycan in the tendon, limits collagen fibril formation and thus directs tendon remodeling due to tensile forces [39]. TNC is a highly conserved family of oligomeric glycoproteins organized in the ECM of vertebrate organisms where its expression suggests tenogenesis in vitro [14, 24]. According to this premise, upregulated expressions of analyzed genes confirmed that the human degenerative tendon matrix induced tenogenesis of BM MSCs.

It has been previously reported that ECM promotes tissue-specific cell differentiation through the preservation of structural, biochemical, and biomechanical motifs found in native tissues [30, 33]. To gain further insight into the tissue-specificity of ECM, relative mRNA expression of ALP and ACAN was investigated. ALP is recognized as an early marker of osteoblast differentiation as it is a by-product of osteoblast activity, thereby suggesting a correlation between increased expression and active bone formation [36]. ACAN is recognized as a hallmark of articular chondrocytes as its covalently attached sulfated glycosaminoglycan [10] chains exert water-attracting characteristics and generate a fixed negative charge of cartilage [8, 31]. CTFG is an extracellular matrix protein recognized for its regulation of diverse biological processes, including angiogenesis, chondrogenesis, and osteogenesis, where its expression drastically increases during skeletal repair or regeneration [3]. It has been validated that CTGF alone is sufficient to differentiate MSCs into fibroblasts, which are ubiquitous cells that comprise the stroma of practically all tissues [21]. Although used as the positive control for tenogenic differentiation of BM MSCs, CTGF also upregulated the relative mRNA expressions of both ALP and ACAN. This observation reflected the limitation of not only the in vitro utilization of CTGF for the tissue-specific differentiation of MSCs but also the development of therapeutic applications regarding regeneration through using only a single growth factor. On the other hand, the human degenerative tendon matrix inhibited chondrogenic differentiation with significantly downregulated expression of ACAN and had a negligible impact on osteogenic differentiation with unchanged expressions of ALP. Therefore, the co-culture between the human degenerative tendon matrix and BM MSCs validated the responsibility of ECM for inducing tissue-specific differentiation of BM MSCs and overcame the limitation of in vitro utilization of a single growth factor for tenogenic differentiation.

When co-cultured with different amounts of tendon matrix, BM MSCs showed a very distinct dose-dependent trend for relative mRNA expression of tenogenic markers of SCX, TNMD, Col I, Col III, DCN, and TNC. Small amounts of tendon matrix, including 0.005 g, 0.01 g, 0.025 g, 0.05 g, 0.1 g, 0.25 g, and 0.5 g, progressively increased the relative mRNA expression of listed tenogenic markers. However, large amounts of tendon matrix, including 1 g and 2 g, progressively decreased the relative expression of the abovementioned tenogenic markers. Furthermore, cellular senescence, which has historically been viewed as permanent proliferative arrest while remaining metabolically active [13, 37], was induced as the amount of tendon matrix exceeded 0.01 g. Since the medium was not supplemented with exogenous factors, such as FBS, and the cells embedded in the tendon matrix had impaired functions due to repetitive freeze-thawing, various regulatory molecules inside the tendon matrix located in the upper chamber of the co-culture system appeared to be solely responsible for inducing cellular senescence along with tenogenic differentiation of BM MSCs. Previous studies regarding the effects of degenerative osteoarthritic ECM on tissue-specific chondrogenesis of MSCs have produced conflicting results on whether the co-cultured ECM promoted or inhibited chondrogenesis [9, 12, 22]. The results from the present study and the conflicting evidence from the previous studies suggest that suitable ratios and adequate amounts of regulatory molecules are required when performing the co-culture system for tissue-specific differentiation of MSCs without inducing cellular senescence.

However, this study had several limitations. First, the cellular senescence induced after an indirect co-culture system between BM MSCs and degenerative tendon matrix was a collateral finding. It may be explained by the use of a degenerative tendon matrix or by the existence of dead cells embedded in the tissue. Because the large amounts of tendon matrix, including 1 g, and 2 g, downregulated the relative mRNA expression responsible for tenogenic differentiation of BM MSCs, senescence assays were not performed accordingly. Further experiments examining the effects of large amounts of degenerative tendon matrix on the cellular senescence of BM MSCs would lead to a better understanding of suitable ratios for effective tissue-specific differentiation. Second, the rotator cuff tendon with a healthy microenvironment of ECM is difficult to obtain due to the degenerative characteristics of the disease. It would have been valuable to investigate the effects of a healthy rotator cuff tendon matrix for sophisticated regulation of the amount of tendon matrix required for tenogenesis of BM MSCs possibly without inducing cellular senescence.

## Conclusion

This study successfully demonstrated tendon ECM-stimulated tenogenesis of BM MSCs through an indirect co-culture system without the use of exogenous growth factors and the alteration of cellular viability. In contrast to the initial hypothesis, the tenogenesis of BM MSCs induced with the degenerative tendon matrix accompanied cellular senescence.

## Data Availability

Not applicable.

## Abbreviation

MSCs: Mesenchymal stem cells
ECM: Extracellular matrix
BM MSCs: Bone marrow mesenchymal stem cells
SCX: Scleraxis
TNMD: Tenomodulin
Col I: Collagen type I
Col III: Collagen type III
DCN: Decorin
TNC: Tenascin-C
ALP: Alkaline phosphatase
ACAN: Aggrecan
SA-βgal: Senescence-associated beta-galactosidase
ISCT: International society for cellular therapy
